# The impact of the evaluations made by Mini-CEX on the clinical competency of nursing students

**DOI:** 10.1186/s12909-022-03667-2

**Published:** 2022-08-20

**Authors:** Sanaz Motefakker, Azam Shirinabadi Farahani, Manijeh Nourian, Maliheh Nasiri, Fatemeh Heydari

**Affiliations:** Shahid Beheshti University of Medical Sciences, Tehran, Iran

**Keywords:** Clinical competency, Evaluation, Mini-Clinical Evaluation Exercise, Nursing student

## Abstract

**Introduction:**

Clinical competency is defined as one’s capabilities in terms of knowledge, attitude and practice, and is a necessity for nursing practice. Evaluation is the inseparable part of the education process, without which the quality of education could not be measured. Considering the characteristics of clinical education and its impact on the clinical competency of students, as well as the importance of making precise evaluations of them using scientific, modern and efficient approaches, this study aims to investigate the impact of the evaluation made using Mini-Clinical Evaluation Exercise on the clinical competency of the nursing students of the School of Nursing and Midwifery Shahid Behesti University.

**Methods:**

This is a quasi-experimental study with a control group conducted on students who were taking courses “Nursing care for a sick child”. The students were classified into the intervention and control groups using complete enumeration. The evaluations were made using Mini-Clinical Evaluation Exercise in the intervention group and the portfolio approach in the control group. The skills regarding patient (mother–child) education, IV therapy and medication were evaluated by checklists.

**Findings:**

The results showed that the mean score of clinical competency in the intervention group was significantly higher than that of the control group.

**Conclusion:**

Considering the improvement of clinical competency in the intervention group, it is recommended to use Mini-Clinical Evaluation Exercise for the evaluation of students.

## Introduction

Nursing students work in complicated environments where technology and practice are constantly changing. Therefore, they need more competence to provide quality care for their care seekers [1]. Learning and adapting to different types of skills and job roles in the education of clinical nursing is difficult, because students have to learn a lot of clinical interventions [2]. Clinical skills are defined as the prudent and consistent use of knowledge, communication and technical skills, clinical talent, emotions and values ​​in the clinical environment, which in turn creates clinical competency [3]. Clinical competency consists of integrated capabilities and abilities in the field of knowledge, attitude and skills that is a prerequisite for nursing practice and includes problem solving skills and the ability to function effectively, which in some specialties, such as nursing, is a requirement to fulfill the job role and maintain the professional position [4]. The lack of clinical competency causes the student to be incompetent in Patient education, performing nursing procedures and performing other hospital duties [5]. Studies have shown that students new to nursing traineeship, do not have the adequate skills and proficiency in clinical settings, despite having a strong theoretical knowledge, and act poorly in the problem-solving process. Thus, one of the most important and challenging issues in clinical skills training and improving clinical competency is the issue of evaluating students [6]. Evaluating students in the form of direct observation in practical and real contexts will ensure students’ ability to face and predict clinical incidents and improve clinical competency regarding the patient’s specific condition [7]. One of the methods of educational evaluation is the Mini Clinical Evaluation Exercise (Mini-CEX), which is a test for evaluating students' clinical skills and giving feedback on their performance at the same time. Mini-CEX offers many advantages. The original purpose of this test is to provide feedback and modify learners’ abilities, although there are numerous reports on its use as a final purpose and for making final decisions regarding learners' performance [8]. It is important to note that this evaluation approach has helped improve student-patient interactions and provide better care services, and has also improved student-evaluator interactions while evaluating clinical skills [5]. In general, students and specialists were positive to very positive about the practicality of the Mini-CEX and about the impact of this assessment format on learning and on professional development [9] In December 2019, COVID-19 emerged and spread rapidly. In many countries, including Iran, in order to contain the outbreak of COVID-19, in-person education in universities became mostly limited and almost suspended [10]. As a result, the presence of nursing students in the patient's bed has decreased and part of the internship courses has been held virtually. Given the importance of clinical education, the promotion of clinical skills and its impact on the clinical competence of nursing students as individuals who will provide patient care in the near future, conducting research to improve clinical competency seems important.

On the other hand, accurate and constructive evaluation of students with appropriate scientific methods such as Mini-CEX can solve the problems of teaching and learning and develop students with more competence.

So far, no study has been conducted in Iran to investigate the effect of Mini-CEX on the clinical competence of nursing students, so the researchers hypothesized that Mini-CEX is effective on the clinical competence of nursing students. The aim of this study was to investigate the effect of Mini-CEX on the clinical competency of nursing students.

### Type of study

This is a quasi-experimental study with a control group and was performed as a single blind. That is, only the researcher himself is aware of the type of intervention and its purpose. One type of evaluation (Mini-CEX or portfolio) was performed in each medical center and all students in one center received the same intervention.

### Research instrument

The instruments used in this research consisted of demographic information questionnaire and short nursing competencies questionnaire. The demographic Information questionnaire was developed by the researcher team and consisted of seven items including age, mean grading in education, gender, marital status, the place of residence, student work experience and working experience in the pediatric departments. The short nursing competencies questionnaire was developed by Watson et al. (2002), with the aim of investigating the clinical competency of nursing students [11], which was reevaluated and edited by the developers in the same year and finalized with 18 items. The questionnaire was scored on a 4-point Likert scale, with 18 and 72 being the minimum and maximum scores, respectively. At the first step, in order to use the instrument, correspondence was exchanged with the developers via e-mail and the permission to use was obtained.

The questionnaire was then translated according to the guidelines made by Wilde et al. (2005). It was translated into Farsi separately by the researcher and another fluent English translator. Efforts were done in order to avoid changes in the meaning, the concept and the level of difficulty of the items. The translated versions were then reviewed by a third translator who had no part in the initial translation process and the final version was prepared. This final version questionnaire was back-translated into English. The final version was sent to the questionnaire developer and approved by them. In order to determine the face validity of the tool, it was examined by 10 students meeting the inclusion criteria and a survey was conducted about vague items. Information was also collected on how to phrase the scale. Content validity was also assessed through a qualitative approach [12, 13]. To this end, 10 experts in the fields of nursing, management and scale development expressed their opinions and suggestions in regard with Farsi grammar, using appropriate and correct words, the correct placement of items and the scoring process. In order to determine the reliability of the questionnaire, the internal consistency reliability was measured through the calculation of Cronbach's alpha after 15 students meeting the inclusion criteria completed the questionnaire. To examine the stability reliability, a test–retest was done and the results were compared. To this end, 15 students meeting the inclusion criteria were selected through random sampling to complete the questionnaires again two weeks later. Then the stability reliability was determined by calculating the intraclass correlation coefficient (ICC). The ICC and Cronbach’s alpha were reported to be 0.99 and 0.88, respectively, both above 0.7 and acceptable.

## Method

Nursing students are taking a clinical nursing course “Nursing care for a sick child “. They learn how to care for sick children. This course is taught by the faculty members of the School of Nursing and Midwifery of Shahid Beheshti University of Medical Sciences. The whole course was performed in the hospital. Due to the prevalence of Covid-19 disease and the unsuitable conditions of the wards for student admission, the duration of this internship was reduced by half. But for more accurate training, students were trained virtually for a week through the LMS system.

Students were trained in pediatric nursing skills and assessed by Mini-CEX in three skills: patient (mother- child) education, IV therapy and medication. In child care, the method of care is family-centered; therefore, patient and family education are one of the most important skills in caring for a sick child. On the other hand, due to the sensitivity of children to the serum and drug levels, teaching the method of calculating and injecting both is very important in children. Therefore, the three skills mentioned; Patient (mother- child) education, IV therapy and medication are the most important skills that a nursing student should learn and practice in caring for sick children. Since the education process is not complete without evaluation, it is essential to have an academic and efficient way to assess these skills in nursing students. The Mini-CEX is a test to assess students' clinical skills and provide feedback on their performance at the same time. The evaluator observes the student during the practice and gives feedback on the performance, so the students understand the mistakes. Mini-CEX is a multi-step test in which students can correct their mistakes in later steps. In the final stage, the total score for the students is calculated. The preparation process of Mini-CEX was as follows: preparation of blueprint, determination of the skills to be assessed, design of evaluation forms, determination the minimum acceptable level of performance in each area, decide on the number and characteristics of evaluators, informing, familiarizing and educating the evaluators and participants, implementing Mini-CEX and examination of the quality of the test held. In the intervention group, the first Mini-CEX test was held on the first day of the trainee program. According to the condition and illness of each patient in hospital ward, a scenario is given to each student that explains the illness and nursing care that is needed such as mother–child education, IV therapy, and medication and the students according to the scenario perform these three skills of (mother–child) education, IV therapy, and medication for the patient. Then the examiner, according to the check list of three skills of patient (mother–child) education, IV therapy, and medication evaluates the skills of students by Mini-CEX in two stages; one stage in the beginning of course and the other in the end of course. These three clinical skills are the most important skills that every nursing student should know and implement for nursing care and there is no need to evaluate all clinical skills in test and in each encounter, we focus on some clinical skills. Every clinical skill had a checklist. In “patient (mother–child) education” checklist these items were mentioned: introducing the student to the mother and child, how to communicate with child according to the age of the child, listen to the mother and child, making eye contact with them, assess the educational needs of mother and child by asking questions, explain the treatments appropriate to the age of the child, explain the treatment to the mother and answering the mother’s questions according to the mother’s literacy. In “IV therapy” checklist these items were mentioned: kardex review, choosing the appropriate serum, calculating the correct electrolyte dose, hand wash, pull the electrolyte correctly from the vial, calculating the number of drops per minute, writing the serum label and sticking it on the serum, establishing the effective therapeutic communication with the child and mother, checking the IV line for signs of phlebitis, checking the IV line for date and ensuring proper serum infusion. In “medication” checklist this items were mentioned: checking medication’s card, choosing the appropriate serum, calculating the correct medicine dose, hand wash, solving the medicine properly, micro set preparation, writing the micro set label and sticking it on the micro set, establishing the effective therapeutic communication with the child and mother, checking the IV line for signs of phlebitis, checking the IV line for date, ensuring proper medicine infusion and pay attention to Drugs’ side effects and recording the medicine’s report. The scenario was developed in order to assess the above skills in the student while answering the relevant questions in the presence of the patient at the ward. The student explained the answers. At the end of the test, the evaluator gave feedback to the student regarding his/her strengths and weaknesses. The feedback was provided verbally by solving the desired scenario about patient and bringing up examples similar to the scenario questions for the student, in order to achieve better understanding. The checklist of the first test in the three skills patient (mother–child) education, IV therapy and medication were filled out in the presence of the student. While filling out the checklist by the evaluator, the student directly figured out the positive and negative points of his/her answers and the reason for the reduced scores in each skill. The test process took 25 min in general, 15 min for the student to respond and 10 min for verbal feedback and the completion of checklists.

**Fig. 1:**
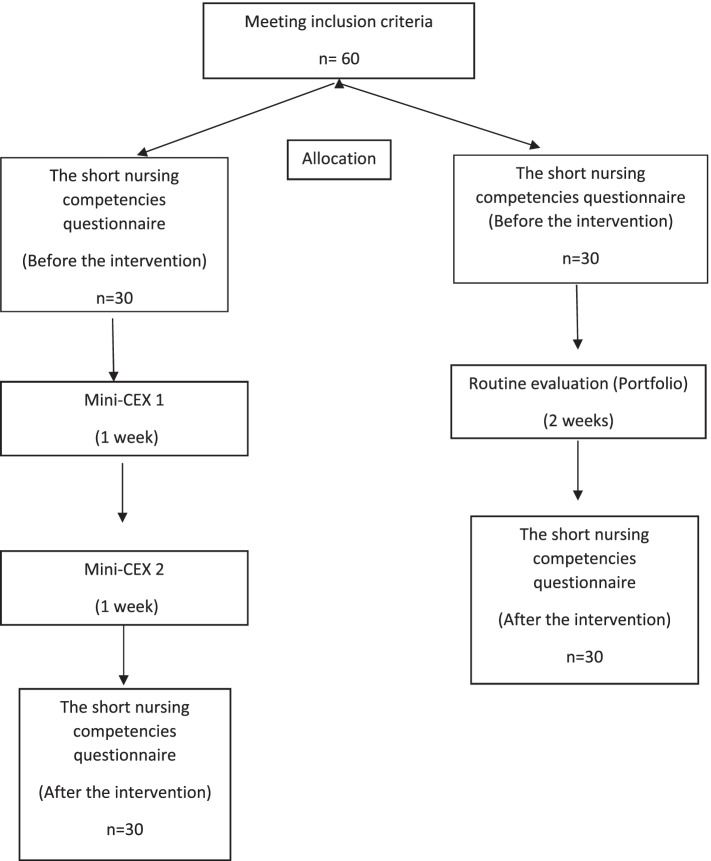
Diagram of CONSORT

At the end of the first test, students completed the demographic Information questionnaire and short nursing competencies questionnaire. The second test was held one week later. During that week, due to the growth of COVID-19 spread and the inappropriate condition of wards for trainees, at the discretion of the pediatric group, students’ training was done virtually through the LMS system, running on Adobe Connect. In virtual education, in addition to common traineeship trainings, the skills which were evaluated in the students were also taught. The second and final test was performed using the same approach. At the end of the second test, the students again completed the short nursing competencies questionnaires and submitted them to the evaluator. The second test defined the final score of the student, which was also considered as the traineeship program’s total score. In the control group, the evaluation of students was done using the common portfolio of the pediatric department of Shahid Beheshti University of Medical Sciences. The portfolio also measures students’ clinical skills in patient (mother- child) education, IV therapy, and medication. On the first day of the traineeship, the portfolios for (mother- child) education, IV therapy and medication were handed over to the students to be completed by the end of the traineeship and submitted on the last day. The students completed the demographic information questionnaire and the short nursing competencies questionnaire. In the control group, due to the pandemic outbreak and inappropriate condition of wards for trainee programs, for one week, students’ training was done virtually through the LMS system, at the discretion of the pediatric group. Virtual education, in addition to the usual training, also focused on teaching the skills which were evaluated in the students. At the end of the traineeship program, the student handed over the relevant portfolio and completed the short nursing competencies questionnaire again (Fig. 1). After the intervention, students were appreciated for participation in the study.

### Statistical methods

The data was analyzed using SPSS19, and descriptive and analytical statistics including Chi-square test, Fisher's exact test, paired t-test, independent t-test and ANCOVA were performed.

### Ethical considerations

This study approved by the ethics committee of School of Nursing and Midwifery, Shahid Beheshti University of Medical Science (IR.SBMU.PHARMACY.REC.1399.145). All methods were performed in accordance with the relevant guidelines and regulations by including a statement in the Declarations section to this effect. All the students participated in research willingly, after signing the written informed consent.

All students who were taking the internship course “Nursing care for a sick child” formed a sample of the study by census. Then they were randomly divided into intervention and control groups. The inclusion criteria consisted of taking the theoretical course of “Nursing care of children” in the School of Nursing and Midwifery of Shahid Beheshti University of Medical Sciences, Tehran, as well as taking this trainee course for the first time and having no working experience in pediatric departments and no history of employment in nursing profession. Exclusion criteria were student absence in each of the evaluation stages.

### Findings

The aim of this study was to investigate the effect of Mini-CEX evaluation on the clinical competency of nursing students.

In this study the mean age of the students was 22.37 in the intervention group, and 22.80 in the control group. Their mean grading in education was 16.45 (out of 20) in the intervention group, and 16.82 (out of 20) in the control group. Table 1 shows some of the demographic variables.

**Table 1:** The demographic information of students participating in the study

**Variable**	**Variable values**	**Intervention group**		**Control group**		***P*-value**
		**Frequency**	**Percentage**	**Frequency**	**Percentage**	
Marital status^a^	Single	29	96.67	28	93.33	0.5
	Married	1	3.33	2	6.67	
Place of residence^b^	Home	21	70	16	53.33	0.184
	Dorm	9	30	14	46.67	
Gender^b^	Female	18	60	17	56.67	0.793
	Male	12	40	13	43.33	

^a^According to Fisher’s exact test

^b^According to Chi-square test

A comparison of the clinical competency scores indicated that the mean score of clinical competency in the intervention and control groups was significantly different before and after the intervention, showing an increase after the intervention (*P* < 0.001). Therefore, ANCOVA was used to compare the mean scores of clinical competency after the intervention in both groups (Table 2).

**Table 2:** A comparison of the mean and the standard deviation of clinical competency scores, before and after the intervention in control and intervention groups

**Group/clinical competency score**	**Intervention**	**Control**	***P*-value**
	**Mean (SD)**	**Mean (SD)**	
Pre-intervention	47.57(0.65)	55/17(1.18)	< 0.001^a^
Post-intervention	64/60(0.94)	54.57(1.14)	< 0.001^b^
Paired t-test results	0.001	0.0006	

^a^According to independent t-test

^b^According to ANCOVA

**Fig. 2:**
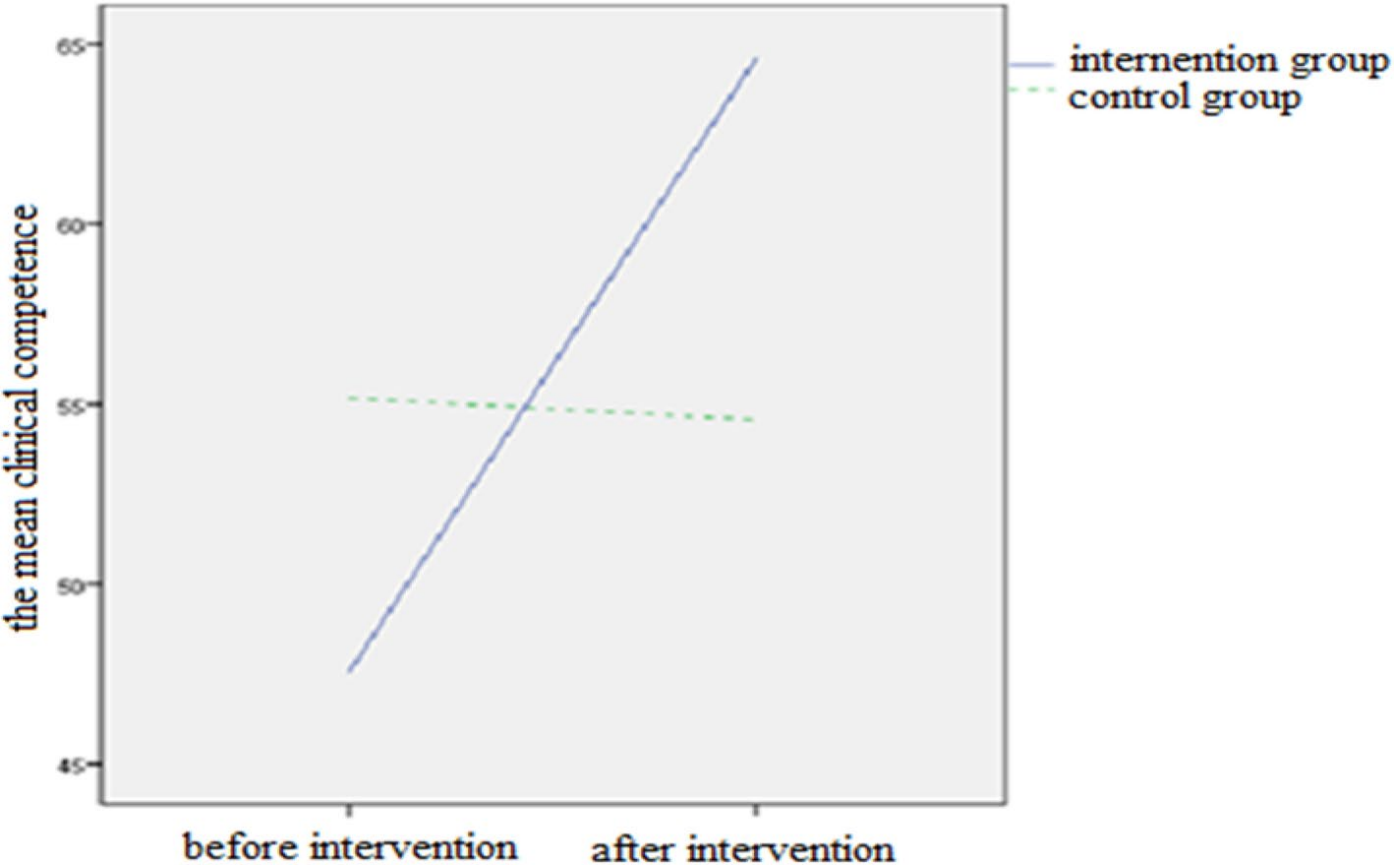
The trend of changes in the mean clinical competence over time for the two intervention and control groups in nursing students

The trend of changes in the mean clinical competence over time for the two interventions and control groups in nursing students is shown in Fig. 2.

## Discussion

The present study was conducted to investigate the impact of Mini-CEX evaluation on the clinical competency of nursing students. The results of this study indicate that Mini-CEX, which was used in the intervention group, can improve the clinical competency of nursing students and the study objectives have been achieved. The study by Moudgil et al., which examined the clinical competency of ophthalmology interns using Mini-CEX and DOPS in on-call rotation, concluded that the Mini-CEX and DOPS evaluations are useful for investigating the clinical competency of interns and have strengthened their clinical skills [14]. A study was conducted by Khalil et al. on the implementation of Mini-CEX as a program for evaluating students’ clinical competency. The results of this study showed that the residents participating in this study had a high level of satisfaction with Mini-CEX evaluation approach and that this approach has improved their clinical skills and is evidently highly accepted among them [15]. The control group, which was evaluated by the portfolio approach, showed that the portfolio does not affect the clinical competency of nursing students. However, there are differences between the results of the present study and other studies. Sedaghat et al., who studied the impact of educational portfolio on the clinical competency of nursing students, showed that the implementation of the educational portfolio has effects on improving the clinical competency of nursing students [16]. This inconsistency may be due to the fact that in the mentioned researches, the portfolio has been examined against the conventional evaluation approaches, but in the present study, the portfolio itself has been considered as a conventional approach and compared to the novel Mini-CEX evaluation method.

By comparing the clinical competency of the nursing students before and after the intervention in the intervention and control groups, the results of this study indicate that the Mini-CEX has been effective on the clinical competency of nursing students. In the studies that have been done so far, the researchers have concluded that the Mini-CEX approach improves clinical competency. According to the trend of changes in the mean clinical competence over time for the two intervention and control groups in nursing students, changes in the control group with portfolio evaluation increased with less slope but changes in intervention group with Mini-CEX evaluation increased with a greater slope and this shows that because Mini-CEX is a new approach to evaluation, it is more effective and productive than the other method of evaluations. The study by Amila et al., which was conducted on nursing students with the aim of investigating the impact of Mini-CEX in achieving clinical competency in clinical examinations, reported an improved clinical competency among nursing students through the use of Mini-CEX evaluation approach [5]. Another study was conducted by Jasemi et al. to investigate the impact of evaluations made by the direct observation of procedural skills (DOPS) and the traditional portfolio method on learning clinical skills among nursing students. The results of this study also showed that using DOPS approach, the mean scores were higher in the intervention group than in the control group, which implemented conventional methods [17], which is consistent with the results of the present study. The results of the above researches show that Mini-CEX evaluation has improved the clinical skills of nursing students, which is in line with the results of the current study.

## Conclusion

The results of the present study showed that Mini-CEX evaluation is effective in the clinical competency of nursing students. The students’ clinical skill evaluation scores have increased significantly during the Mini-CEX evaluation, and this approach has effectively improved the clinical competency of the nursing students. The implementation of this evaluation approach leads to the improvement of the clinical skills of nursing students, which ultimately results in training capable nurses with favorable clinical competency, and improves the quality of nursing care. Therefore, this indicates the need to pay attention to modern evaluation approaches, especially Mini-CEX approach as an effective factor in improving the clinical competency of nursing students, and the necessity of implementing this approach to evaluate nursing students and even the students of other medical disciplines in medical universities.

### Implication of practice

Clinical competence is one of the most important issues in nursing students. Students can provide better care for sick children in the future as nurses and caregivers if they have the desired clinical competence. Given that childcare is always “family-centered care”, nurses' clinical competence can support the whole family through their involvement in care.

### Research limitations

Since this study was conducted during COVID-19 pandemic, the number of students was lower than usual and the duration of the traineeship was reduced, and part of the students’ training was done online through LMS system.

### Future research suggestions

In this study, the impact of Mini-CEX evaluation on the clinical competency of nursing students was investigated. It is recommended to compare this approach with other evaluation approaches in future studies. Suggested studies are as follows:

1. Comparing the impact of Mini-CEX and logbook evaluation on the clinical competency of nursing students
2. Comparison of the effect of Mini-CEX and portfolio evaluation on the clinical competency of nursing students
3. Comparison of the effect of Mini-CEX and OSCE evaluation on the clinical competency of nursing students

## Data Availability

The datasets generated during and/or analyzed during the current study are not publicly available due to the datasets generated during and / or analyzed during the current study are not publicly available due to the lack of completion of the manuscript review and acceptance process, But are available from the corresponding author on reasonable request.
